# A 2-year prognostic assessment of patients with coronary borderline lesions based on three-vessel quantitative flow ratio and the development and internal validation of a risk prediction nomogram

**DOI:** 10.3389/fcvm.2026.1907097

**Published:** 2026-10-12

**Authors:** Hua Bai, Rigun A, Gang Zhang, Zhijie Guo, Dahai Bing, Yajiang Zhang, Peng Liu, Xi Liu

**Affiliations:** 1 Affiliated Ordos Clinical College of Inner Mongolia Medical University, Ordos City, Inner Mongolia, China; 2 Department of Cardiovascular Medicine, Ordos Central Hospital, Ordos, Inner Mongolia, China; 3 Graduate School of Baotou Medical College, Inner Mongolia University of Science and Technology, Baotou, China; 4 Department of Cardiovascular Medicine, Second Affiliated Hospital of Harbin Medical University, Harbin, Heilongjiang, China

**Keywords:** borderline coronary lesions, LASSO regression, major adverse cardiovascular events (MACE), nomogram, three-vessel quantitative flow ratio (3V-QFR)

## Abstract

**Objective:**

To investigate the prognostic value of three-vessel quantitative flow ratio (3V-QFR) in patients with borderline coronary lesions (QFR 0.75–0.80) over a 2-year period; to identify key variables using LASSO regression; and to construct and internally validate a personalized nomogram model for predicting major adverse cardiovascular events (MACE) over 2 years.

**Methods:**

This single-center retrospective cohort study included patients with coronary artery disease who were treated at Ordos Central Hospital from January 2021 to December 2024, underwent coronary angiography revealing at least one borderline lesion in a major coronary artery, and received a QFR assessment. Patients were followed for up to 24 months after discharge through outpatient visits, telephone follow-up, and electronic medical-record review. The primary endpoint was defined as the occurrence of MACE during the 2-year follow-up period. Restricted cubic spline (RCS) analysis was used to explore the nonlinear relationship between 3V-QFR and the risk of MACE. Least absolute shrinkage and selection operator (LASSO) penalized regression evaluated 19 prespecified candidate predictors using 10-fold cross-validation and the one-standard-error rule. A nomogram was constructed based on the selected factors, and the model’s discriminatory power, consistency, and clinical net benefit were evaluated using receiver operating characteristic (ROC) curves, calibration curves, and decision curve analysis (DCA). Internal validation used 1,000 bootstrap resamples that repeated the complete preprocessing, variable-selection, and model-refitting pipeline.

**Results:**

A total of 138 patients with borderline lesions were enrolled (33 in the MACE group and 105 in the non-MACE group). Among 33 first MACE events, 20 were ischemia-driven revascularizations, 9 were non-fatal myocardial infarctions, and 4 were all-cause deaths. RCS analysis showed an overall association between lower 3V-QFR and higher MACE risk, whereas evidence for nonlinearity was not statistically strong; multivariable regression supported 3V-QFR as an independent predictor. LASSO regression identified four optimal predictors of 2-year MACE through 10-fold cross-validation: 3V-QFR, age, diabetes, and NT-proBNP levels. The final model included 3V-QFR, age, diabetes, and NT-proBNP. The apparent AUC was 0.869 (95% CI 0.802–0.936), and the optimism-corrected AUC was 0.842 (95% CI 0.771–0.913); calibration and decision-curve findings were considered exploratory.

**Conclusion:**

Lower 3V-QFR was associated with higher 2-year MACE risk in this retrospective development cohort. The 3V-QFR-based nomogram showed promising internal performance and may support exploratory risk stratification; independent external and prospective validation is required before clinical use.

## 1 Introduction

The precise assessment and treatment of coronary atherosclerotic heart disease are currently key priorities in the field of cardiovascular medicine. In clinical practice, borderline lesions identified during coronary angiography typically refer to those with a visual stenosis of 40% to 70%; such lesions have long represented a “gray area” in decision-making regarding interventional procedures (1, 2). Relying solely on the degree of anatomical stenosis often fails to accurately determine whether a lesion has caused true myocardial ischemia. Blindly performing percutaneous coronary intervention (PCI) not only fails to improve long-term patient outcomes but may also increase the risk of in-stent thrombosis and bleeding, while imposing an unnecessary financial burden on patients; conversely, overly conservative management may lead to the overlooking of high-risk ischemic lesions (3, 4).

The fractional flow reserve (FFR) technique serves as the “gold standard” for evaluating coronary vascular function and addresses this issue. The 2018 European Society of Cardiology guidelines recommend FFR as a Class IA recommendation for guiding the management of borderline lesions in stable coronary artery disease (5). However, the standardized procedure for FFR is relatively complex. The intraoperative use of vasodilators such as adenosine can easily lead to serious adverse reactions in patients, including palpitations, hypotension, and even shock. Furthermore, the high cost of the pressure-sensing guidewires required for the procedure limits its widespread clinical application. Quantitative Flow Ratio (QFR), an emerging derivative technology of FFR, has undergone continuous development and refinement since 2014 and has been validated in numerous large-scale, prospective, multicenter clinical trials, such as FAVOR Pilot (6), FAVOR II China (7), FAVOR II Europe-Japan (8), and WIFI II (9). These studies demonstrate that QFR exhibits a high degree of consistency with FFR in the diagnosis of borderline lesions and treatment decision-making. Furthermore, the 2024 guidelines published by the European Society of Cardiology recommend QFR as a Class IB recommendation for guiding the management of coronary artery disease (10). More recent diagnostic work has supported QFR assessment in intermediate lesions (11), while FAVOR III China reported favorable clinical outcomes with QFR-guided lesion selection at 1 and 2 years (12, 13).

However, current studies on QFR often focus on the diagnostic and prognostic value of stenosis in a single vessel, with few studies evaluating patients by combining the left anterior descending artery, circumflex artery, and right coronary artery. Yet, coronary artery disease is often a diffuse, systemic condition involving multiple vessels. Physiological impairment in a single vessel may not be sufficient to trigger a cardiovascular event, but the cumulative burden of overall myocardial ischemia may be a key determinant of a patient's long-term prognosis (14, 15). Currently, there are few reports on the prognostic value of three-vessel quantitative flow ratio (3V-QFR), defined as the sum of LAD, LCX, and RCA QFR and therefore integrating the physiological status of all three vessels, in patients with borderline coronary lesions. Related global QFR and coronary-physiology studies have also been reported (16, 17). Contemporary work has further linked residual QFR, plaque characteristics, and final postprocedural QFR with subsequent clinical outcomes (18–20).

Therefore, this study aims to explore the nonlinear dose-response relationship between 3V-QFR and the risk of MACE in patients with borderline coronary lesions. Concurrently, by incorporating advanced statistical dimension-reduction techniques such as LASSO regression and combining them with traditional clinical risk factors, we constructed and validated an individualized risk prediction nomogram model (21, 22) to explore its potential for risk stratification in patients with borderline lesions; external validation is required before clinical use.

## 2 Research methods

### 2.1 Study population and data collection

This single-center retrospective cohort study reviewed consecutive patients with coronary artery disease who underwent coronary angiography (CAG) at our hospital between January 2021 and December 2024 and were found to have at least one major coronary artery (anterior descending artery, circumflex artery, or right coronary artery) with a borderline lesion (visual diameter stenosis between 40% and 70%, and a corresponding QFR value between 0.75 and 0.80). Patients were followed for up to 24 months after discharge and were censored at the last confirmed contact or administrative data cutoff. Patients were classified into a MACE group and a non-MACE group based on the occurrence of major adverse cardiovascular events (MACE) at the 2-year follow-up endpoint. Baseline demographic characteristics (e.g., age, sex), medical history (e.g., hypertension, diabetes), and laboratory test results (e.g., NT-proBNP, LDL-C) were systematically collected. Both acute coronary syndrome and stable coronary artery disease presentations were eligible. For multiple lesions in one vessel, distal vessel-level QFR represented the cumulative functional effect. Of 249 screened patients, 29 met prespecified clinical exclusion criteria, 55 had inadequate angiography or non-calculable QFR, and 27 had incomplete data or unavailable follow-up; 138 were included (Supplementary Figure S1). Angiography, laboratory values, index-hospitalization PCI, and discharge medications were treated as baseline information; follow-up was scheduled at 1, 3, 6, 12, and 24 months by outpatient visit or telephone and supplemented by medical-record, readmission, and death-record review.

### 2.2 3V-QFR measurement and definition of endpoints

The 3-vessel QFR was calculated based on two standard two-dimensional coronary angiography images with an angle difference of ≥25°, using three-dimensional vascular reconstruction and computational fluid dynamics algorithms. 3V-QFR is defined as the sum of the QFR values of the three major epicardial vessels—the left anterior descending artery (LAD), the left circumflex artery (LCX), and the right coronary artery (RCA)—where at least one vessel has a borderline lesion (QFR 0.75–0.80), while the remaining vessels are either borderline lesions (QFR 0.75–0.80) or normal vessels (QFR > 0.80). QFR was calculated offline from archived angiograms using the AngioPlus Pro II platform (Shanghai Pulse Medical Technology, Shanghai, China); the exact archived software build could not be reliably retrieved. Angiography was acquired at 15 frames/s after intracoronary nitrate administration, using two projections separated by at least 25° and iohexol injection at approximately 3–4 mL/s for the left coronary artery and 2–3 mL/s for the right coronary artery. Adequate contrast filling, clear lumen borders, minimal overlap or foreshortening, and limited motion were required. All three major vessels had to be technically analyzable. Two trained interventional cardiologists with at least 5 years of coronary angiography/PCI experience and more than 100 prior QFR analyses independently reviewed each analysis; disagreements were resolved by a senior cardiologist.

The primary endpoint was the first MACE occurring after discharge and within 24 months: all-cause death; non-fatal myocardial infarction defined according to the universal definition; or unplanned ischemia-driven PCI or CABG prompted by ischemic symptoms and/or objective evidence of ischemia. Planned or staged procedures related to the index hospitalization were not counted as MACE. Two cardiologists independently adjudicated events using outpatient notes, telephone records, readmission records, procedural reports, laboratory/electrocardiographic findings, and death documentation; disagreement was resolved by a third cardiologist.

### 2.3 Statistical analysis

All statistical analyses were performed using R (version 4.3.1). Continuous variables were expressed as mean ± standard deviation or median (interquartile range) based on the results of normality tests; comparisons between groups were performed using the independent samples t-test or the Mann–Whitney U test. Categorical variables were expressed as frequency (percentage) and analyzed using the chi-square test or Fisher's exact test. Missing values occurred for hs-CRP (5/138, 3.6%), NT-proBNP (4/138, 2.9%), eGFR (2/138, 1.4%), LDL-C (3/138, 2.2%), and HbA1c (3/138, 2.2%). Under a missing-at-random assumption, multiple imputation by chained equations generated 10 datasets (23, 24). The observed outcome and all candidate predictors were included in the imputation model, but outcome values were not imputed; selection and fitting were repeated across datasets and estimates were pooled using Rubin's rules.

To eliminate the interference of multicollinearity in the model, Pearson's correlation coefficient was used to assess the correlation among continuous variables. Considering the potential complex dose-response relationship between physiological indicators and clinical prognosis, this study employed a restricted cubic spline with 4 nodes (25) to evaluate the association between 3V-QFR and MACE risk (26). Knots were placed at the 5th, 35th, 65th, and 95th percentiles (approximately 2.52, 2.60, 2.66, and 2.75), with 2.63 as the reference; the model adjusted for age, diabetes, and NT-proBNP and tested overall association and nonlinearity. Because exact event times were not consistently available, 24-month MACE was modeled as a binary outcome, which does not incorporate event timing or censoring.

During the feature selection stage, to limit model complexity relative to the number of events, LASSO penalized regression was employed for dimensionality reduction (27). Binomial deviance was calculated using 10-fold cross-validation, and the optimal regularization parameter (*λ*) was determined based on the stricter“one-standard-error”criterion. The core variables identified by LASSO and clinically recognized risk factors were incorporated into a multivariate logistic regression model to extract adjusted odds ratios and Z-statistics for quantifying feature importance. Finally, an individualized nomogram was constructed based on the core independent predictors (28), and the model's discriminatory power, consistency, and clinical net benefit were comprehensively evaluated using ROC curves, Bootstrap internal resampling (1,000 times) calibration curves (29), and decision curve analysis (30). A two-sided *P*-value < 0.05 was considered statistically significant. Nineteen candidates were evaluated: age, sex, smoking, hypertension, diabetes, prior myocardial infarction, prior PCI, clinical presentation, LVEF, NT-proBNP, hs-CRP, eGFR, LDL-C, HbA1c, platelet count, LAD stenosis, LCX stenosis, RCA stenosis, and 3V-QFR. Continuous variables were standardized, binary variables were coded 0/1, and multicategory variables used indicator terms. The selected values were *λ*min = 0.0396 and *λ*1se = 0.0692; the 1-SE rule retained 3V-QFR, age, diabetes, and NT-proBNP.

A nomogram was constructed from the four retained predictors. Apparent AUC 95% CIs were obtained using DeLong's method. Internal validation used 1,000 bootstrap resamples of the complete modeling pipeline; in each resample, optimism was the difference between performance in the bootstrap sample and performance of the refitted model in the original data. The mean optimism was subtracted from apparent performance. Percentile bootstrap intervals were used for optimism-corrected AUC and Brier score; Wald intervals were used for the calibration slope. Sensitivity and specificity intervals used the Wilson method, and predictive-value intervals used a logit-based method. Calibration was assessed using the Brier score, calibration intercept, calibration slope, and a bootstrap-corrected curve with patient-level risk-distribution marks. The optimal probability threshold was derived in the development cohort by maximizing the Youden index and was considered exploratory. Decision-curve analysis compared the model with treat-all and treat-none strategies. To compare aggregate with vessel-specific physiology on the same analytical scale, four exploratory covariate-matched logistic models were fitted; each included age, diabetes, NT-proBNP, and one QFR measure (LAD QFR, LCX QFR, RCA QFR, or 3V-QFR). Model AUCs were estimated using DeLong's method, and paired AUC differences versus the 3V-QFR model were evaluated using 5,000 patient-level bootstrap resamples. Two-sided *P* < 0.05 indicated statistical significance.

## 3 Study results

### 3.1 Differences in baseline characteristics and 3V-QFR distribution

The cohort comprised 138 patients, including 33 with MACE and 105 without MACE. Expanded baseline comparisons are shown in Table 1. Mean 3V-QFR was numerically lower in the MACE group, while the other consistently recorded characteristics were generally comparable between groups (Figure 1A). Pearson correlation analysis among the selected continuous variables is shown in Figure 1B. Lower 3V-QFR strata were associated with a greater frequency of multivessel physiological impairment, supporting its interpretation as an aggregate physiological-burden measure (Supplementary Table S1).

**Figure 1:**
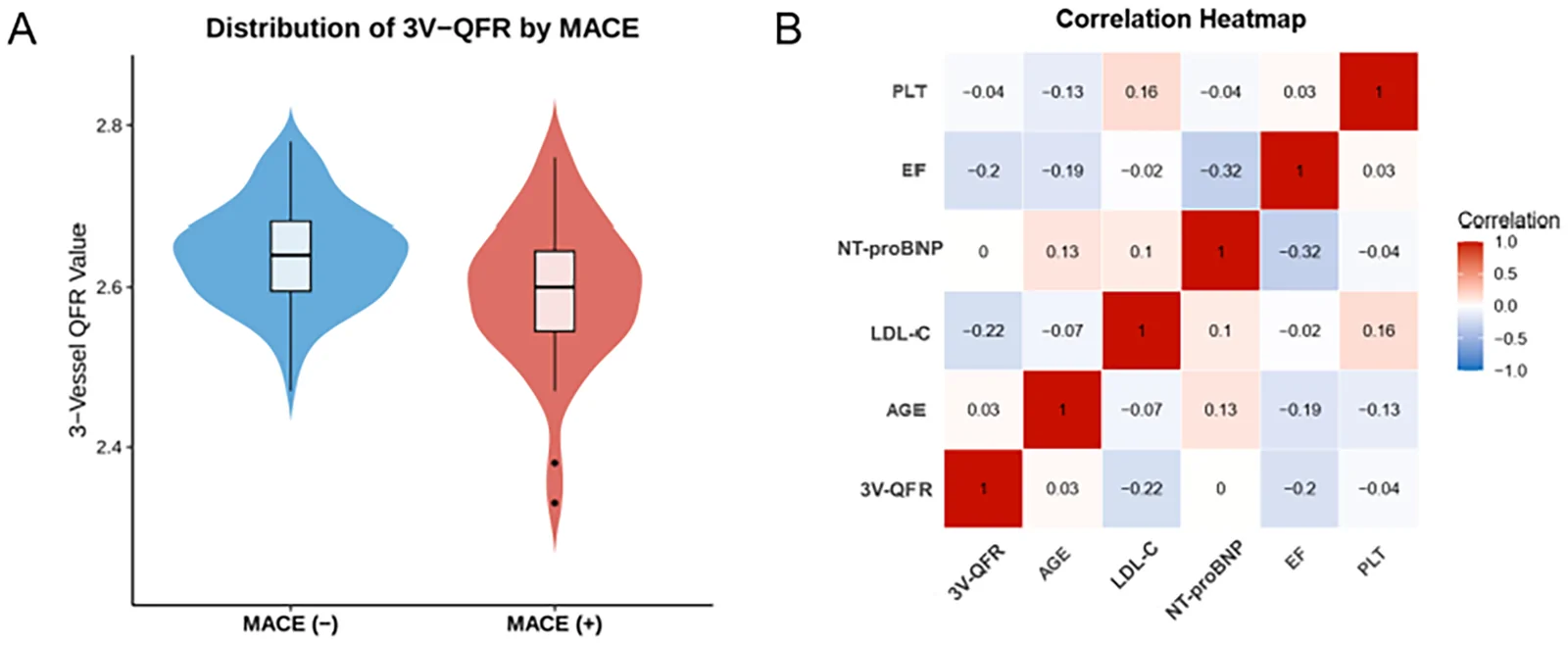
**(A)** violin plot: comparison of 3V-QFR distributions between the two patient groups; **(B)** Pearson correlation heatmap: correlation matrix among baseline indicators.

**Table 1:** Baseline characteristics of the study cohort.

Characteristic	Overall (*N* = 138)	Non-MACE (*n* = 105)	MACE (*n* = 33)	*P* value
Age, years	60.4 ± 11.2	60.2 ± 11.2	61.0 ± 11.4	0.705
LAD QFR	0.859 ± 0.075	0.859 ± 0.075	0.858 ± 0.076	0.990
LCX QFR	0.885 ± 0.082	0.889 ± 0.082	0.875 ± 0.083	0.412
RCA QFR	0.883 ± 0.086	0.886 ± 0.087	0.875 ± 0.085	0.498
3V-QFR	2.627 ± 0.075	2.633 ± 0.074	2.608 ± 0.074	0.089
Male sex, *n* (%)	114 (82.6%)	87 (82.9%)	27 (81.8%)	0.891
Hypertension, *n* (%)	85 (61.6%)	60 (57.1%)	25 (75.8%)	0.055
Diabetes mellitus, *n* (%)	36 (26.1%)	27 (25.7%)	9 (27.3%)	0.859
Current smoking, *n* (%)	89 (64.5%)	68 (64.8%)	21 (63.6%)	0.906
PCI during index hospitalization, *n* (%)	93 (67.4%)	69 (65.7%)	24 (72.7%)	0.453

Data are mean ± SD or *n* (%). Comparisons used Welch's *t* test, chi-square test, or Fisher exact test, as appropriate. LAD, left anterior descending artery; LCX, left circumflex artery; RCA, right coronary artery; QFR, quantitative flow ratio; PCI, percutaneous coronary intervention.

Among the 33 first MACE events, 20 were ischemia-driven revascularizations (60.6%), 9 were non-fatal myocardial infarctions (27.3%), and 4 were all-cause deaths (12.1%). Patients with more than one event were counted once according to the first event.

### 3.2 Nonlinear threshold effects and independent predictive value of 3V-QFR for MACE risk

In the fully adjusted restricted cubic spline model (age, diabetes, and NT-proBNP), the overall association between 3V-QFR and MACE was significant (Poverall <0.001), whereas evidence for nonlinearity was not statistically strong (Pnonlinearity = 0.150). The curve suggested increasing risk at lower 3V-QFR values, but no definitive clinical threshold was inferred because of the limited number of events and the narrow observed 3V-QFR range (Figure 2A). Multivariable analysis supported 3V-QFR as an independent predictor after adjustment for the other retained variables (Figure 2B).

**Figure 2:**
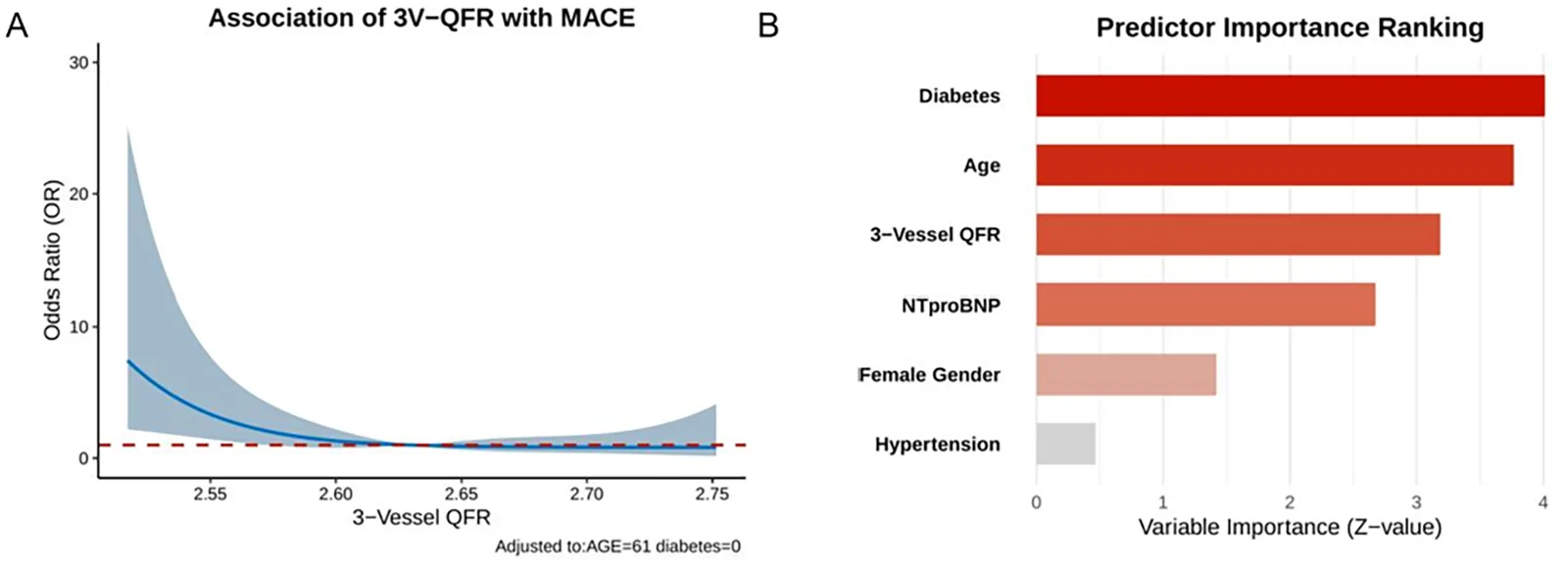
**(A)** restricted cubic spline relationship between 3V-QFR and MACE risk, adjusted for age, diabetes, and NT-proBNP (reference 3V-QFR = 2.63; poverall <0.001; Pnonlinearity = 0.150); **(B)** predictor-importance plot for the multivariable model.

### 3.3 Feature selection and risk scorecard construction using LASSO regression

Among 19 prespecified candidate variables, 10-fold cross-validated LASSO using the 1-SE rule (*λ*1se = 0.0692; log *λ* = −2.671) retained 3V-QFR, age, diabetes, and NT-proBNP. The final model therefore contained four predictors, corresponding to 8.25 events per predictor (33/4), compared with 1.74 events per candidate variable before selection (33/19). A nomogram was constructed from the final model (Figure 3).

**Figure 3:**
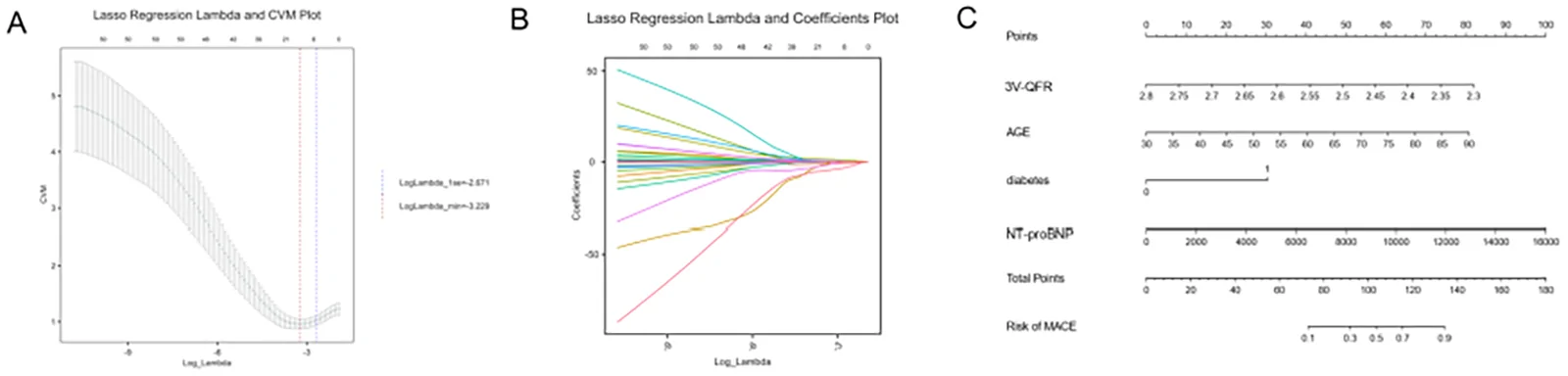
**(A)** ten-fold cross-validation curve for LASSO; **(B)** coefficient paths and selection at the 1-SE value (*λ*1se = 0.0692; log *λ* = −2.671); **(C)** nomogram for 2-year MACE risk.

### 3.4 Validation of the predictive model's multidimensional performance

The apparent AUC was 0.869 (95% CI 0.802–0.936). Mean bootstrap optimism was 0.027, giving an optimism-corrected AUC of 0.842 (95% CI 0.771–0.913). At the exploratory data-derived probability threshold of 0.355, sensitivity was 81.8% (95% CI 65.7–91.9%), specificity 80.0% (95% CI 71.3–86.5%), PPV 56.3% (95% CI 42.3–69.3%), and NPV 93.3% (95% CI 86.4–96.9%). The apparent and optimism-corrected Brier scores were 0.124 and 0.138 (95% CI 0.102–0.174), respectively; the optimism-corrected calibration intercept was 0.012 (95% CI −0.315 to 0.339) and slope 0.930 (95% CI 0.690–1.170), indicating acceptable calibration with some uncertainty and possible mild overfitting. The risk-distribution marks are shown in Figure 4B; because only 33 events occurred, numerical bootstrap intervals were emphasized rather than an unstable pointwise confidence ribbon. The nomogram provided greater net benefit than treat-all and treat-none strategies at threshold probabilities of approximately 5%–85% (Figure 4C). A positive classification would prompt closer follow-up, reinforcement of guideline-directed preventive therapy and adherence, and selective ischemia reassessment rather than automatic revascularization. The threshold and DCA findings are exploratory.

**Figure 4:**
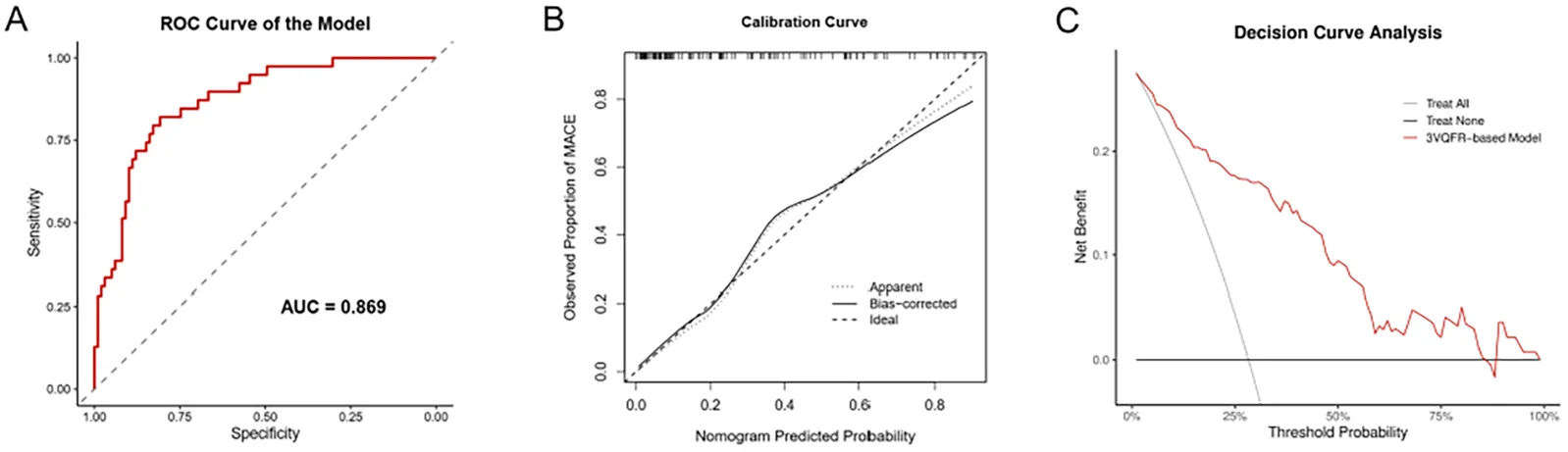
**(A)** ROC curve for the four-predictor model (apparent AUC 0.869, 95% CI 0.802–0.936; optimism-corrected AUC 0.842, 95% CI 0.771–0.913); **(B)** apparent and bootstrap bias-corrected calibration curves with patient-level risk-distribution marks; **(C)** decision-curve analysis. Model net benefit exceeded treat-all and treat-none strategies over approximately 5–85% threshold probability.

In exploratory covariate-matched analyses, the model incorporating age, diabetes, NT-proBNP, and 3V-QFR had the highest apparent AUC (0.869, 95% CI 0.802–0.936), compared with otherwise identical models incorporating LAD QFR (0.838, 95% CI 0.762–0.913), LCX QFR (0.832, 95% CI 0.757–0.908), or RCA QFR (0.863, 95% CI 0.800–0.925). The AUC differences versus the 3V-QFR model were 0.032 (*P* = 0.231), 0.037 (*P* = 0.037), and 0.007 (*P* = 0.755), respectively. Thus, 3V-QFR yielded the highest numerical discrimination, but evidence of improvement was statistically significant only in comparison with LCX QFR (Supplementary Table S2).

## 4 Discussion

This retrospective cohort study evaluated aggregate coronary physiology in patients with borderline lesions. Lower 3V-QFR was associated with higher MACE risk after multivariable adjustment, and a four-predictor nomogram showed promising internally validated discrimination and acceptable calibration. In exploratory covariate-matched analyses, the model incorporating 3V-QFR had the highest apparent AUC (0.869), compared with otherwise identical models incorporating LAD QFR (0.838), LCX QFR (0.832), or RCA QFR (0.863). The improvement was statistically significant only versus the LCX-QFR model and not versus the LAD- or RCA-QFR models (Supplementary Table S2). These findings support further evaluation of aggregate physiology but do not establish general superiority or clinical effectiveness.

### 4.1 Physiological assessment of borderline lesions and the significance of total ischemic burden

Conventional wisdom holds that when coronary FFR or QFR falls within the narrow “gray zone” of 0.75–0.80, there is high individual heterogeneity regarding whether the lesion induces severe myocardial ischemia, whether aggressive intervention is warranted, and whether conservative management is appropriate (31). For this borderline population, this study employed the comprehensive functional indicator 3V-QFR to assess the total myocardial ischemic burden. Our spline analysis suggested increasing risk as 3V-QFR decreased; however, the limited event count and restricted 3V-QFR range preclude a firm threshold claim. This result should be considered hypothesis-generating. Prior studies have also examined global three-vessel QFR and the interaction of coronary physiology with systemic factors (16, 17, 36). Recent studies have further evaluated QFR-guided lesion selection, residual and postprocedural QFR, and QFR assessment of nonculprit lesions in multivessel disease across different clinical settings (11–13, 18–20, 37).

### 4.2 Advantages of machine learning in predictive model development

Real-world clinical datasets often contain interrelated clinical and biochemical variables, which can increase instability in an unpenalized multivariable model. In this study, 19 candidate variables were considered relative to 33 events. LASSO was used for simultaneous shrinkage and selection before conventional multivariable logistic regression (28, 32). The final model retained four predictors (8.25 events per predictor); repeating selection and refitting within 1,000 bootstrap samples estimated optimism, although residual overfitting remains possible.

### 4.3 Incremental predictive value of NT-proBNP

In addition to classic cardiovascular risk factors (age and diabetes), LASSO regression incorporated NT-proBNP into the optimal predictive system for patients with borderline lesions. This suggests that for patients with borderline lesions (QFR 0.75–0.80), ischemia in some patients may already be sufficient to cause transient or subclinical diastolic dysfunction or increased ventricular wall tension, thereby stimulating tension-sensitive mechanisms in cardiomyocytes and leading to mild release of NT-proBNP (33). This is consistent with previous findings that “brain natriuretic peptide levels are independently associated with coronary artery severity and poor prognosis in stable coronary heart disease” (34). The combination of 3V-QFR and NT-proBNP enables a dual-dimensional assessment ranging from the level of coronary artery hemodynamics to the extent of myocardial damage.

### 4.4 Limitations

This study has certain limitations. First, as a single-center retrospective study, the sample size (*n* = 138) is relatively small, particularly given the low number of MACE events, which may have limited statistical power to some extent. Second, although the model underwent rigorous internal validation using Bootstrap, it lacks validation in an external independent cohort—a critical hurdle that all machine learning predictive models must overcome for true clinical translation (35). Multicenter prospective external validation is therefore required before clinical use. Furthermore, while the 24-month follow-up period in this study reflects mid-term outcomes, it remains insufficient for assessing the long-term (e.g., 5-year) prognostic value of borderline lesions. Future prospective studies with longer follow-up periods are needed to validate the predictive value of 3V-QFR for long-term MACE. With 19 candidates and 33 events (1.74 events per candidate), residual overfitting is possible despite LASSO and full-pipeline bootstrap validation; the four-predictor model had 8.25 events per predictor. Exact event times were not consistently available, so binary logistic regression could not account for event timing or censoring. Formal observer reproducibility statistics and complete presentation-subtype counts were unavailable. Baseline treatment variables may be affected by treatment confounding or mediation. The risk threshold, vessel-level comparison, and decision-curve findings are exploratory.

## Data Availability

The original contributions presented in the study are included in the article/Supplementary Material, further inquiries can be directed to the corresponding authors.
