# Supplementary material for: A 2-year prognostic assessment of patients with coronary borderline lesions based on three-vessel quantitative flow ratio and the development and internal validation of a risk prediction nomogram
Source: Front Cardiovasc Med. 2026 Oct 12;13:1907097. doi: 10.3389/fcvm.2026.1907097 (PMC13696331; doi:10.3389/fcvm.2026.1907097)
Supplement: Supplementary file 1 [file Datasheet1.docx]

**Supplementary Material**

**Supplementary Figure S1. Participant flow diagram**

**Supplementary Table S1. Baseline characteristics by empirical 3V-QFR stratum**

| **Characteristic** | **Overall (N = 138)** | **Low <2.60 (n = 44)** | **Intermediate 2.60–2.65 (n = 44)** | **High ≥2.66 (n = 50)** | **P value** |
| --- | --- | --- | --- | --- | --- |
| 3V-QFR | 2.63 [2.58, 2.68] | 2.56 [2.53, 2.58] | 2.62 [2.62, 2.64] | 2.69 [2.67, 2.73] | <0.001 |
| Age, years | 61 [53, 68] | 62 [52, 65] | 61 [55, 71] | 60 [53, 68] | 0.373 |
| LAD QFR | 0.87 [0.78, 0.92] | 0.86 [0.79, 0.90] | 0.89 [0.78, 0.92] | 0.80 [0.78, 0.95] | 0.693 |
| LCX QFR | 0.91 [0.79, 0.96] | 0.86 [0.78, 0.92] | 0.90 [0.79, 0.95] | 0.96 [0.92, 0.97] | <0.001 |
| RCA QFR | 0.92 [0.78, 0.96] | 0.81 [0.76, 0.90] | 0.92 [0.79, 0.95] | 0.94 [0.92, 0.97] | <0.001 |
| Male sex, n (%) | 114 (82.6%) | 37 (84.1%) | 36 (81.8%) | 41 (82.0%) | 0.952 |
| Hypertension, n (%) | 85 (61.6%) | 29 (65.9%) | 26 (59.1%) | 30 (60.0%) | 0.772 |
| Diabetes mellitus, n (%) | 36 (26.1%) | 17 (38.6%) | 9 (20.5%) | 10 (20.0%) | 0.071 |
| Smoking, n (%) | 89 (64.5%) | 29 (65.9%) | 26 (59.1%) | 34 (68.0%) | 0.648 |
| Index-hospitalization PCI, n (%) | 93 (67.4%) | 31 (70.5%) | 32 (72.7%) | 30 (60.0%) | 0.368 |
| ≥2 vessels with QFR ≤0.80, n (%) | 4 (2.9%) | 4 (9.1%) | 0 (0.0%) | 0 (0.0%) | 0.019 |

Continuous variables are median [IQR] and categorical variables are n (%). Groups are empirical descriptive strata and are not validated treatment thresholds. The finding that physiological impairment in ≥2 vessels occurred only in the lowest 3V-QFR stratum supports the construct of aggregate physiological burden. LAD, left anterior descending artery; LCX, left circumflex artery; RCA, right coronary artery; PCI, percutaneous coronary intervention.

**Supplementary Table S2. Exploratory comparison of covariate-matched models incorporating individual-vessel QFR or 3V-QFR**

| **Covariate-matched model** | **AUC (95% CI)** | **AUC difference vs 3V-QFR model (95% CI)** | **P value** |
| --- | --- | --- | --- |
| Age + diabetes + NT-proBNP + LAD QFR | 0.838 (0.762–0.913) | 0.032 (−0.021 to 0.106) | 0.231 |
| Age + diabetes + NT-proBNP + LCX QFR | 0.832 (0.757–0.908) | 0.037 (0.001 to 0.096) | 0.037 |
| Age + diabetes + NT-proBNP + RCA QFR | 0.863 (0.800–0.925) | 0.007 (−0.040 to 0.061) | 0.755 |
| Age + diabetes + NT-proBNP + 3V-QFR | 0.869 (0.802–0.936) | Reference | — |

All four models included age, diabetes, and NT-proBNP and differed only in the QFR measure entered. AUC confidence intervals were obtained using DeLong's method. Differences are calculated as the AUC of the 3V-QFR model minus that of the comparator model; 95% CIs and P values for paired differences were obtained from 5,000 patient-level bootstrap resamples. The 3V-QFR model had the highest apparent AUC. The difference was statistically significant only versus the LCX-QFR model; these post hoc comparisons are exploratory and were not used for variable selection.
